# Nailing vs. plating in comminuted proximal ulna fractures – a biomechanical analysis

**DOI:** 10.1186/s12891-020-03637-z

**Published:** 2020-09-17

**Authors:** Johannes Christof Hopf, Tobias Eckhard Nowak, Dorothea Mehler, Charlotte Arand, Dominik Gruszka, Ruben Westphal, Pol Maria Rommens

**Affiliations:** 1 Department of Orthopedics and Traumatology, University Medical Center, Langenbeckstraße 1, 55131, Mainz, Germany; 2 Institute of Medical Biostatistics, Epidemiology and Informatics (IMBEI), University Medical Center, Langenbeckstraße 1, 55131 Mainz, Germany

**Keywords:** Comminution, Nailing, Proximal ulna, Biomechanical study

## Abstract

**Background:**

Comminuted proximal ulna fractures are severe injuries with a high degree of instability. These injuries require surgical treatment, usually angular stable plating or double plating is performed. Nailing of proximal ulna fracture is described but not performed regularly. The aim of this study was to compare a newly developed, locked proximal ulna nail with an angular stable plate in an unstable fracture of the proximal ulna. We hypothesize, that locked nailing of the proximal ulna will provide non-inferior stability compared to locked plating.

**Methods:**

A defect fracture distal to the coronoid was simulated in 20 sawbones. After nailing or plate osteosynthesis the constructs were tested in a servo-pneumatic testing machine under physiological joint motion (0°-90°) and cyclic loading (30 N – 300 N). Intercyclic osteotomy gap motion and plastic deformation of the constructs were analyzed using micromotion video-analysis.

**Results:**

The locked nail showed lower osteotomy gap motion (0.50 ± 0.15 mm) compared to the angular stable plate (1.57 ± 0.37 mm, *p* < 0.001). At the anterior cortex the plastic deformation of the constructs was significantly lower for the locked nail (0.09 ± 0.17 mm vs. 0.39 ± 0.27 mm, *p* = 0.003). No statistically significant differences were observed at the posterior cortex for both parameters.

**Conclusions:**

Nail osteosynthesis in comminuted proximal ulna fractures shows lower osteotomy gap motion and lower amount of plastic deformation compared to locking plate osteosynthesis under laboratory conditions.

## Background

Comminuted proximal ulna fractures are severe injuries and can be combined with additional bony or ligamentous injuries. In the treatment of these lesions, surgical intervention is usually required to restore an anatomical reduction and allow early mobilization like described for complex Monteggia fractures [1–3]. Especially in case of a comminuted proximal ulna fracture the osteosynthesis must provide a high amount of stability to allow bony healing. Liu et al. postulated, that especially Monteggia fractures are often accompanied with comminution of the proximal ulna fracture [4].

For the proximal ulna fracture, a locking compression plate is favored in most of the cases, especially for a fracture distal to the coronoid [5, 6]. Due to the complex anatomy of the proximal ulna and its thin soft tissue coverage, some disadvantages of this surgical technique like cutaneous complications and a high rate of secondary procedures are described [7, 8]. Intramedullary implants are available for fracture fixation of the proximal ulna and show promising biomechanical and clinical results, but are not used regularly in clinical practice [9, 10]. These implants can theoretically reduce disadvantages and complications of plate osteosynthesis due to a less invasive approach and better biomechanical properties [11, 12].

The aim of this study is to compare a newly developed proximal ulna nail with locked compression plating in a comminuted proximal ulnar fracture with a high degree of instability. In our previous biomechanical study, we showed non-inferiority of the newly developed nail in a simple wedge fracture of the proximal ulna with applied fracture compression [13]. We hypothesize, that the proximal ulna nail also offers a non-inferior biomechanical stability compared to locked plating in highly unstable proximal ulna fractures under laboratory conditions.

## Methods

### Materials

This study is a biomechanical comparison of a new locked intramedullary nail and a locked plate in a defect fracture model of the proximal ulna. The biomechanical test setup simulates the tendon forces of the brachialis and the triceps muscles under cyclic loading and under imitation of elbow motion.

**Fig. 1:**
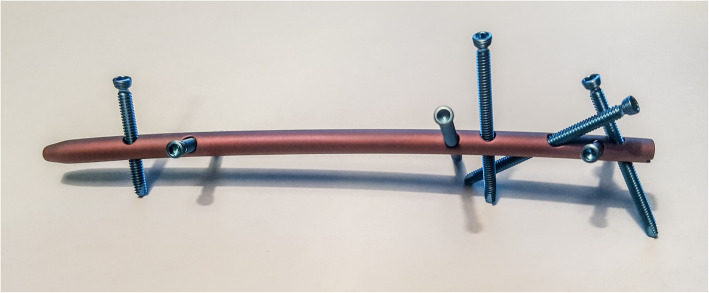
Photo of the nail in anterior-posterior direction with inserted locking screws

Ten pairs of large left 4th generation composite ulnae (#3426, Sawbones® Pacific Research Laboratories, Vashon Island, USA) were used for the biomechanical testing. A standardized defect osteotomy of the proximal ulna distal to the coronoid was simulated in all bones. The performed 10 mm defect osteotomy simulated a comminuted proximal ulna fracture at the junction of the metaphysis and diaphysis of the proximal ulna. Ten sawbones were stabilized with the newly developed locked nail, which is manufactured by Medin a. s. (Nové Město na Moravě, Czech Republic). The nail is made of forged titanium with a diameter of 5 mm, a length of 120 mm and a radial bending of 9°. Figure 1 shows a photo of the nail in anterior-posterior direction with inserted locking screws.

**Fig. 2:**
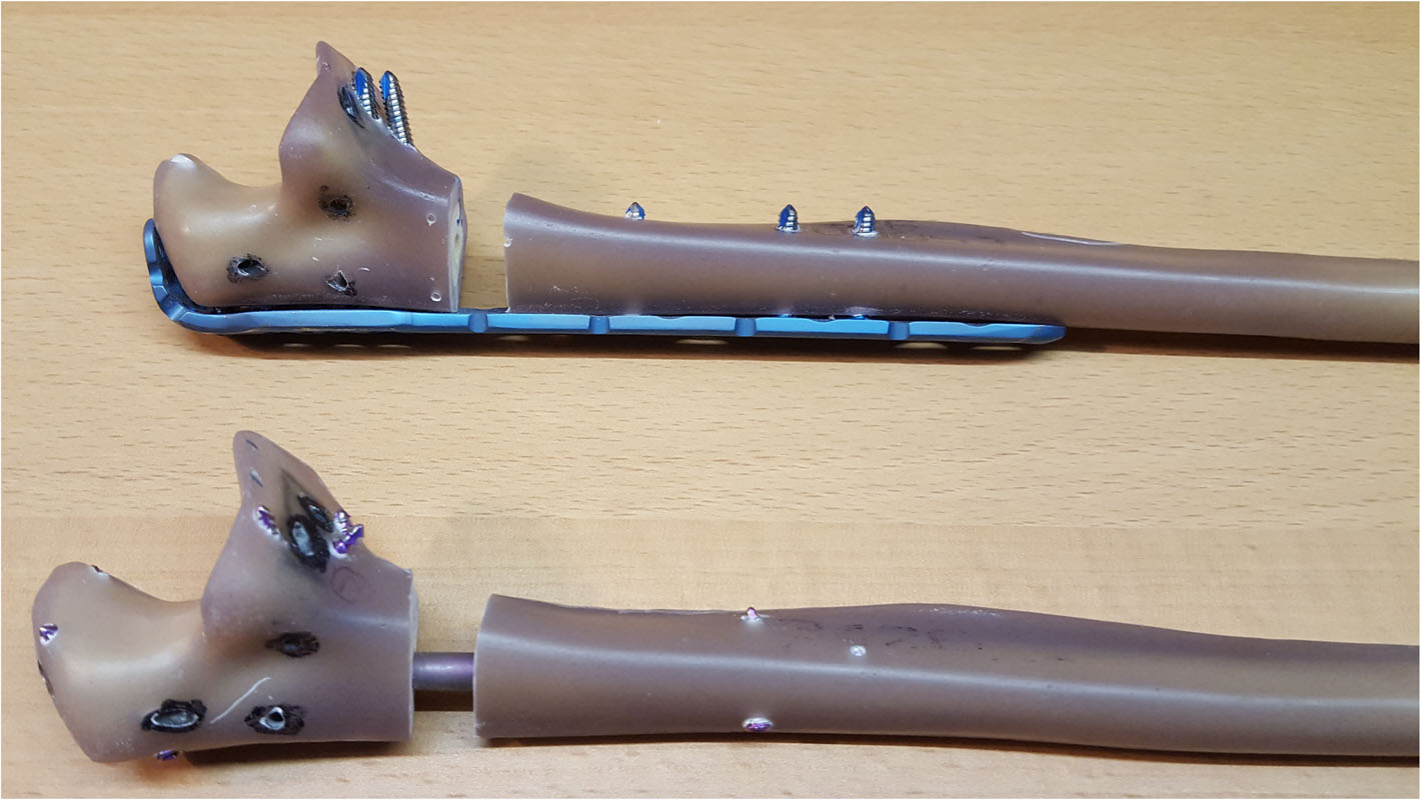
Stabilized sawbones with a dorsal angular stable plate and a locked nail (lateral view)

Another ten sawbones were stabilized with a straight locking plate with a length of 144 mm (Medin a. s.). The two distal holes were not used to simulate a plate with a length of 120 mm for comparison with the 120 mm nail. All twenty fractures were stabilized as a bridging osteosynthesis in a reproducible technique with seven screws in similar screw position in the nail and the plate samples (Fig. 2). All constructs underwent X-ray examinations in two planes for verification of correct screw insertion.

### Test setup

**Fig. 3:**
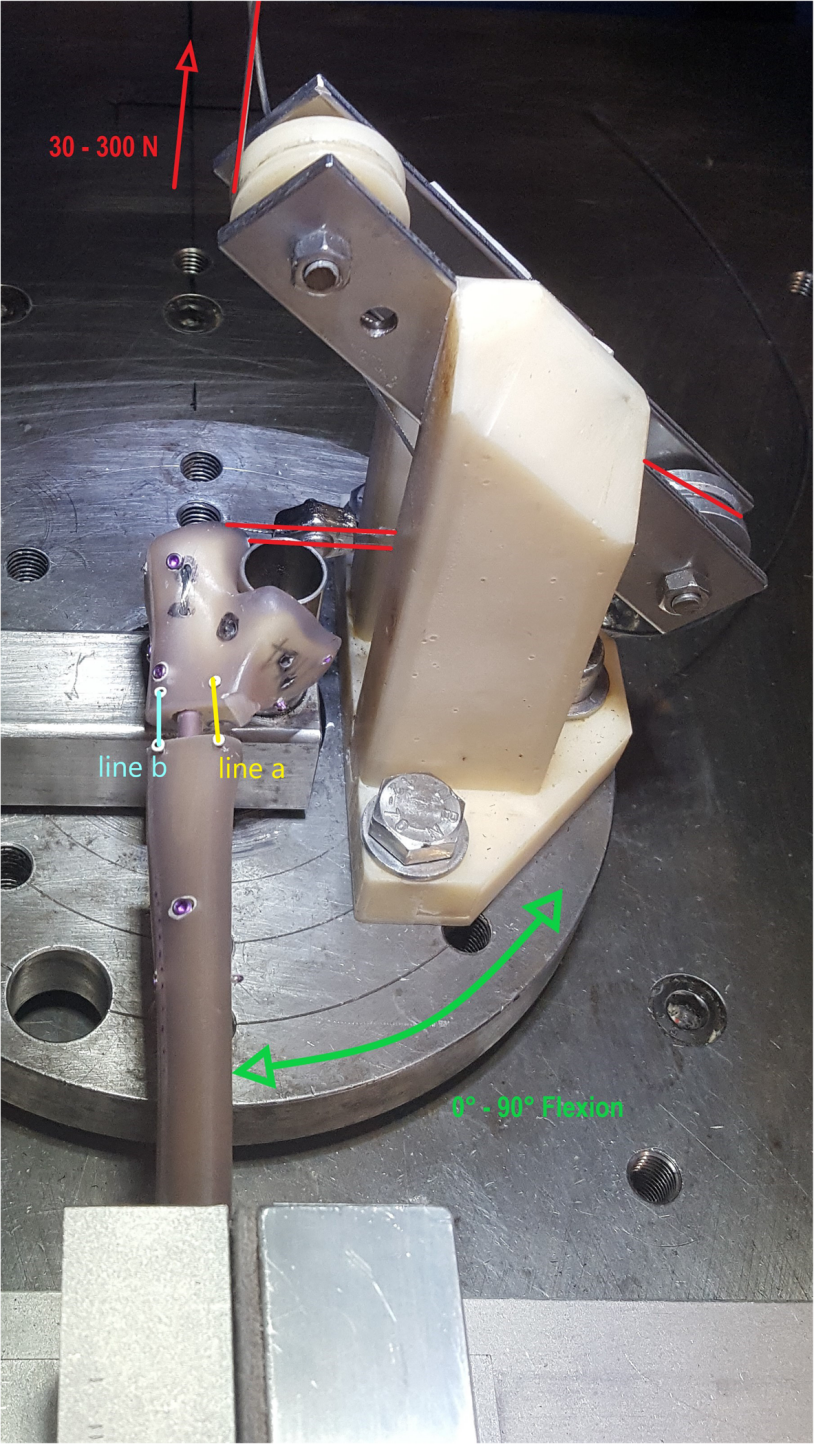
Test set-up with mounted sawbone after nail osteosynthesis and 1.2 mm wire cable for simulation of pulling on the triceps tendon. The direction of the pulling apparatus is highlighted with in red color, the motion of the rotating platform is visualized in green color. The measured distances between the optic markers are highlighted blue (line a: anterior measurement) and yellow (line b: posterior measurement)

**Fig. 4:**
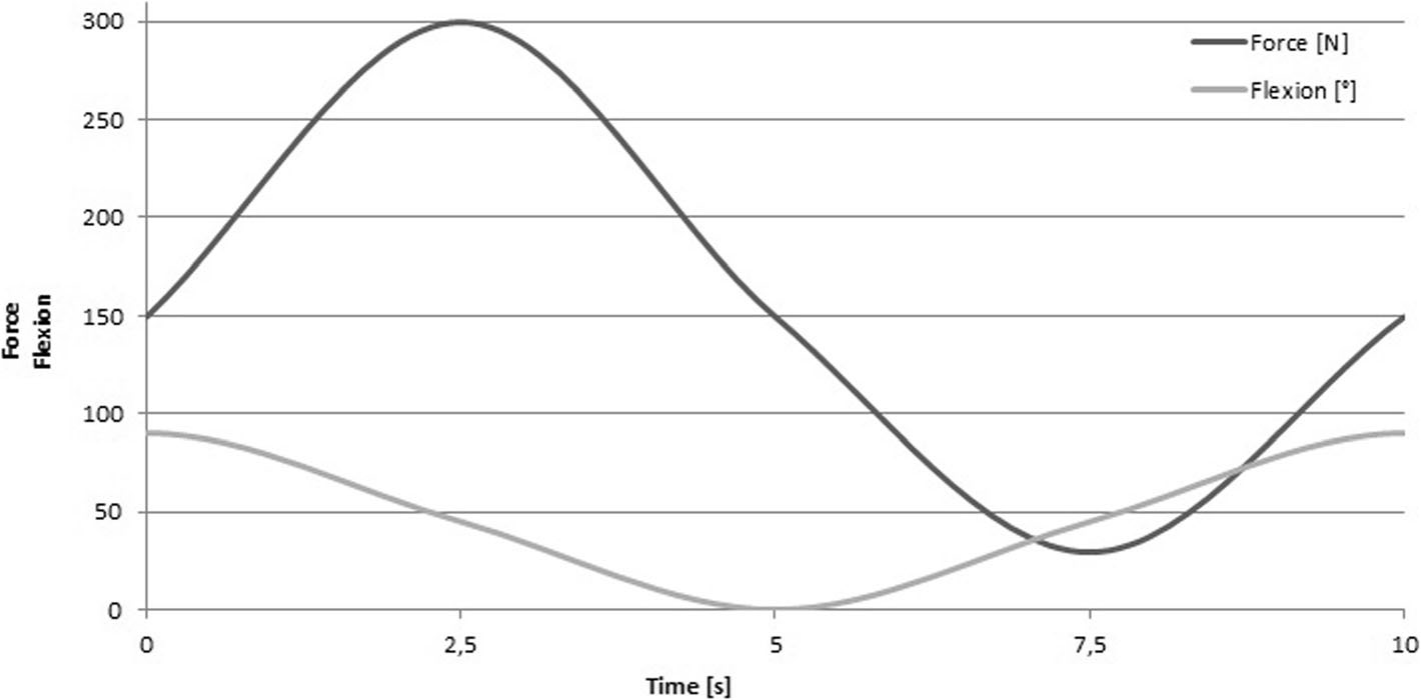
Phase shift of both pneumatic engines during a test cycle. Both engines are synchronized with a phase shift for elbow flexion and applied force.©Springer Nature / European Journal of Trauma and Emergency Surgery

The sawbones were mounted with the distal ulna in a special clamp and the humeral trochlea was imitated by a metal pipe. A 1.2 mm wire cable was mounted to the sawbone through predrilled canals and released at the insertion of the triceps and afterwards the brachialis tendon. The drilling was done under image intensifier to prevent interaction of the wire cable and the implants.A force-controlled servo-pneumatic testing machine (SincoTec, Clausthal-Zellerfeld, Germany) was used for cyclic loading with a maximum force of 300 N and a frequency of 0.1 Hz for 608 cycles. The number of cycles was chosen as a compromise between testing time and validity of the results and for better comparison with the previous study [13]. In all cases we initially performed 304 cycles of triceps pulling followed by 304 cycles of brachialis pulling. With a rotating platform, which worked independently to the linear pulling apparatus, elbow motion between full extension and 90° of flexion was simulated. The pulling direction was adjusted to match the anatomic axis of the triceps and brachialis tendons using a lever with two bearing pulleys. The 1.2 mm wire cable was mounted to the linear pneumatic engine of the testing machine. Figure 3 shows the test setup for triceps pulling with a clamped sawbone. The direction of the wire cable for triceps pulling and the motion of the rotating platform are visualized in Fig. 3. Now, cyclic loading was applied synchronously by the linear and the rotating engine. Both engines worked with a phase shift to mimic the physiological kinetic of the elbow joint, where maximum force can be generated in a mid-flexion position [14–16]. During each test cycle the rotation changed between 0° and 90° of flexion and the pulling force changed between 30 N and 300 N (Fig. 4) [13]. Maximum force of 300 N was chosen following values of activities of normal daily living and were applied in approximately 45° of flexion [15, 16].

### Variables

Two parallel optic marker sets (anterior and posterior) were each placed on the proximal and distal fragment near the fracture gap. The micromotion analysis was performed with a video camera system (CSC-795, Pacific Europe, Waterloo, Belgium) with an optical resolution of 540 × 540 pixels and an accuracy of 2/100 mm. To quantify the intercyclic osteotomy gap motion (elastic deformation) of the implants the distance between the anterior (line a) and posterior (line b) markers were measured during the test procedure (Fig. 3). According to Osterhoff et al. intercyclic osteotomy gap motion was defined as the maximum distance of both lines measured during all 608 test cycles, including the reversible cyclic motions [17]. During the first four test cycles the mean distance between the markers was measured for a baseline value. The plastic deformation of the construct was defined as the difference of the marker distance after 608 cycles and the baseline value, to provide information about the loosening of the constructs during cyclic loading.

### Data analysis and statistics

With an alpha level set at 0.05, the sample size of ten pair of sawbones was calculated for a power (1 − β) of 0.8 and an effect size of 1.4. Micromotion analysis was done with SIMI motion 2D (SIMI, Unterschleißheim, Germany) and the data was statistically analyzed using a two-sided Mann-Whitney Rank Sum Test for independent samples with the statistical software SigmaStat (Systat Software GmbH, Erkrath, Germany). Graphic illustration of elastic and plastic deformation of both implants was done with boxplots.

## Results

The test process included 608 test cycles for each sample and was completed by all constructs. None of the constructs failed or showed macroscopic changes of implant position after the testing process. Table 1 shows the micromotion analysis of the nail and plate groups regarding osteotomy gap motion and plastic deformation of the constructs.

**Table 1:** Results of the micromotion analysis of nails and plates (mean value and standard deviation) and *p*-value after Mann-Whitney Rank Sum Test

Parameters	Nail	Plate	***p***-value
**Anterior cortex**			
Osteotomy gap motion [mm]	0.50 ± 0.15	1.57 ± 0.37	< 0.001
Plastic Deformation of constructs [mm]	0.09 ± 0.17	0.39 ± 0.27	0.003
**Posterior cortex**			
Osteotomy gap motion [mm]	0.37 ± 0.06	0.43 ± 0.14	0.571
Plastic Deformation of constructs [mm]	0.06 ± 0.05	0.07 ± 0.07	0.970

### Intercyclic osteotomy gap motion

**Fig. 5:**
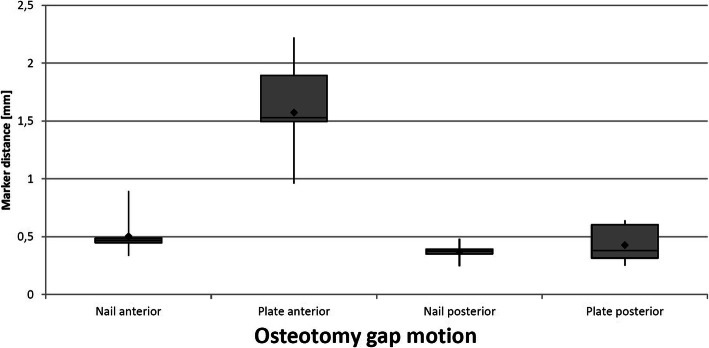
Graphic illustration of osteotomy gap motion of both implants, separated for anterior and posterior cortex in a box-plot

In our test setup the nail constructs show a lower osteotomy gap motion (0.50 ± 0.15 mm) compared to the plate constructs (1.57 ± 0.37 mm) at the anterior cortex. The difference between both groups is statistically significant (*p* < 0.001). At the posterior cortex the nail constructs show a lower osteotomy gap motion as well but without statistical significance (nail 0.37 ± 0.06 mm, plate 0.43 ± 0.14 mm, *p* = 0.571). Figure 5 shows a graphic representation of both groups regarding osteotomy gap motion.

### Plastic deformation of the constructs

**Fig. 6:**
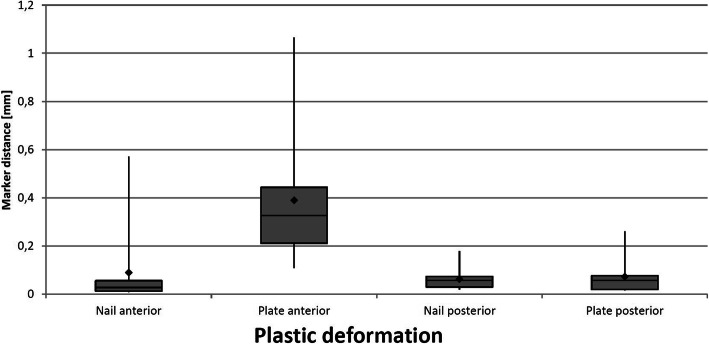
Graphic illustration of the plastic deformation of both constructs, separated for anterior and posterior cortex in a box-plot

According to Osterhoff et al. plastic deformation of the constructs was defined as the difference in displacement after 608 cycles compared to the baseline value of cycle 1–4 [17]. The analysis of the plastic deformation of the constructs shows lower displacement values for the nail constructs with a statistical significance only at the anterior cortex (nail 0.09 ± 0.17 mm, plate 0.39 ± 0.27 mm, *p* = 0.003). At the posterior cortex no relevant difference between the two constructs could be detected (nail 0.06 ± 0.05 mm, plate 0.07 ± 0.07 mm, *p* = 0.970). Figure 6 shows the plastic deformation of both constructs in comparison.

## Discussion

Our results confirm our hypothesis of sufficient primary stability of the nail in highly unstable fracture patterns of the proximal ulna in laboratory conditions. Locked nailing of unstable proximal ulna fractures provides low osteotomy gap motion and a low rate of plastic deformation compared to locked plating. Referring to the results of our previous study, the type of fracture influences the amount of osteotomy gap motion, without an influence on the plastic deformation of the constructs (Table 2) [13]. The lower primary stability (osteotomy gap motion) of the defect osteotomy compared to the wedge fracture can be explained by the missing fragment contact. In contrast, the construct showed no signs of increased osteotomy gap motion at the end of the testing procedure, which shows a sufficient implant stability for the postoperative rehabilitation period.

**Table 2:** Comparison of the micromotion analysis of the nail in a defect situation and wedge fracture (mean value and standard deviation) and *p*-value after Mann-Whitney Rank Sum Test

Parameters	Wedge-facture	Defect situation	***p***-value
**Anterior cortex**			
Osteotomy gap motion [mm]	0.29 ± 0.13	0.50 ± 0.15	< 0.001
Plastic Deformation of constructs [mm]	0.08 ± 0.06	0.09 ± 0.17	0.089
**Posterior cortex**			
Osteotomy gap motion [mm]	0.27 ± 0.11	0.37 ± 0.06	0.014
Plastic Deformation of constructs [mm]	0.05 ± 0.03	0.06 ± 0.05	0.910

To verify the biomechanical advantages and to evaluate benefits and drawbacks in clinical situations, further clinical studies with the new implant are needed.

The standard treatment option for a comminuted proximal ulna fracture is locked plating [18]. Restoration of the complex anatomy of the proximal ulna is the key factor for a good functional outcome according to many authors [18–20]. The quality of reduction of proximal ulna fractures also depends on the implant used for fixation. Anatomically preshaped plates are available especially for restoration of the posterior and varus angulation of the proximal ulna [21, 22]. Double plating is an alternative fixation method and promising results were reported with this technique [23, 24]. In a recent biomechanical study the comparison of a dorsal LCP (locking compression plate) with medial and lateral double plating showed comparable results [25].

Intramedullary fixation of proximal ulna fractures was described by Thompson and Hamilton in 1950 [26]. In the past decades several intramedullary implants were developed, mostly for the treatment of olecranon fractures or osteotomies [27–30]. Despite reports of good biomechanical and clinical results this technique is not established in clinical practice [7, 9, 11, 12].

If the fracture type and localization allow intramedullary fixation, we prefer this technique due to biomechanical advantages and the possibility of a less invasive approach. Intramedullary nailing has become an established treatment option for many kinds of fractures. In our previous study we presented a new intramedullary nail for fracture fixation of proximal ulna fractures. Our biomechanical analysis of the new implant showed a superior stability compared to locked plating [13]. In case of simple fractures anatomic reduction and fracture compression can be achieved with the novel implant to achieve high stability for primary bone healing. In comminuted fractures the nail can be used as a bridging construction possibly combined with a closed reduction technique, which supports callus formation and secondary bone healing. The results of this study showed sufficient biomechanical results under laboratory conditions for the new implant also in highly unstable fractures. Especially at the anterior cortex the intramedullary nail has biomechanical advantages over the dorsally located plate, which increase with the amount of instability of the fracture pattern due to the missing anterior buttressing of the fragments. The minimally invasive approach is an option for appropriate fractures, but should never impair anatomic reduction of the fragments if achievable.

Our test setup was successfully used in our previous study with a proximal ulna wedge fracture as well as in dynamic testing of olecranon fractures in cadaveric bones [13, 31]. The dynamic setup with cyclic loading under continuous elbow motion with a phase shift of both forces imitates the dominant flexor and extensor muscles. Due to technical reasons it was not possible to simulate both forces simultaneously, so we decided to load the constructs consecutively. Compared to other biomechanical studies where static setups were used, the dynamic test setup is closer to physiological conditions in our opinion [32, 33]. The parameters of the test machine have been defined to imitate the forces in the humeroulnar joint during the rehabilitation process [15, 16].

No biomechanical test setup is capable of a perfect reproduction of physiological conditions in the postoperative period after osteosynthesis of proximal ulna fractures, which is definitely a limitation of our study as well. Another restriction of our study is the use of composite synthetic bones instead of cadaveric bones. Biomechanical results with human cadaveric bones are closer to the clinical practice, but struggle with a higher diversity of the bone quality. We used sawbones to guarantee comparable bone quality of all samples [34, 35].

## Conclusions

In this biomechanical analysis we evaluated the osteotomy gap motion and plastic deformation of a new developed locked nail in comparison to locked dorsal plating. Our results show superior stability of the locked nail compared to angular stable plating. The fracture pattern has an influence on osteotomy gap motion but does not affect the plastic deformation of the constructs. We conclude that the new nail provides adequate stability for surgical fixation of highly unstable proximal ulna fractures in laboratory conditions. Clinical studies are needed to verify our results in physiological conditions.

## Data Availability

The datasets used and/or analyzed during the current study are available from the corresponding author on reasonable request.

## Abbreviations

MD: Doctor of Medicine
DIPL-ING: Graduate engineer
IMBEI: Institute of Medical Biostatistics, Epidemiology and Informatics
N: Newton
Hz: Hertz
a. s.: Akciová společnost (joint-stock company)
LCP: Locking Compression Plate
